# Both intraoperative medial and lateral soft tissue balances influence intraoperative rotational knee kinematics in bi-cruciate stabilized total knee arthroplasty: A retrospective investigation

**DOI:** 10.1186/s12891-021-04709-4

**Published:** 2021-09-27

**Authors:** Kentaro Takagi, Hiroshi Inui, Shuji Taketomi, Ryota Yamagami, Kenichi Kono, Kohei Kawaguchi, Shin Sameshima, Tomofumi Kage, Sakae Tanaka

**Affiliations:** Department of Orthopaedic Surgery, Faculty of Medicine, The University of Tokyo, 7-3-1 Hongo, Bunkyo-ku, Tokyo, 113-0033 Japan

**Keywords:** Bi-cruciate stabilized, Medial soft tissue balance, Lateral soft tissue balance, Tibial internal rotation, Total knee arthroplasty

## Abstract

**Background:**

Tibial internal rotation following total knee arthroplasty (TKA) is important in achieving favorable postoperative clinical outcomes. Studies have reported the effect of intraoperative soft tissue balance on tibial internal rotation in conventional TKA, no studies have evaluated the effects of soft tissue balance at medial or lateral compartments separately on tibial internal rotation in bi-cruciate stabilized (BCS) TKA. The purpose of this study was to clarify the relationship between medial or lateral component gaps and rotational knee kinematics in BCS TKA.

**Methods:**

One hundred fifty-eight knees that underwent BCS TKA were included in this study. The intraoperative medial and lateral joint laxities which was defined as the value of component gap minus the thickness of the tibial component were firstly divided into two groups, respectively: Group M-stable (medial joint laxity, ≤ 2 mm) or Group M-loose (medial joint laxity, ≥ 3 mm) and Group L-stable (lateral joint laxity, ≤ 3 mm) or Group L-loose (lateral joint laxity, ≥ 4 mm). And finally, the knees enrolled in this study were divided into four groups based on the combination of Group M and Group L: Group A (M-stable and L-stable), Group B (M-stable and L-loose), Group C (M-loose and L-stable), and Group D (M-loose and L-loose). The intraoperative rotational knee kinematics were compared between the four Groups at 0°, 30°, 60°, and 90° flexion, respectively.

**Results:**

The rotational angular difference between 0° flexion and maximum flexion in Group B at 30° flexion was significantly larger than that in Group A at 30° flexion (*p < 0.05). The rotational angular difference between 30° flexion and maximum flexion in Group B at 30° flexion was significantly larger than that in Group D at 30° flexion (*p < 0.05). The rotational angular differences between 30° or 90° flexion and maximum flexion in Group B at 60° flexion were significantly larger than those in Group A at 60° flexion (*p < 0.05).

**Conclusion:**

Surgeons should pay attention to the importance of medial joint stability at midflexion and lateral joint laxities at midflexion and 90° flexion on a good tibial internal rotation in BCS TKA.

## Introduction

Total knee arthroplasty (TKA) is a reliable procedure for relieving pain or restoring function for progressed knee joint destruction. Many factors affect postoperative pain or function following TKA [1, 2]. As for knee kinematics, the knees following TKA do not always show a medial pivot pattern but a lateral or less pivot pattern [3], although normal or osteoarthritic knees present a medial pivot pattern [4, 5]. The medial pivot pattern following TKA is important in achieving good postoperative patient-reported outcome measures (PROMs) [6]. Furthermore, some studies have reported that the amount of intraoperative tibial internal rotation, which was determined between 60° and 120° flexion or between 60° and 135° flexion, was positively correlated with postoperative knee flexion angle in cruciate-retaining (CR) or posterior-stabilized (PS) TKA, respectively [7, 8]; therefore, for surgeons, understanding how to acquire a favorable tibial internal rotation following TKA is important.

Recently, a bi-cruciate stabilized (BCS) knee system, Journey II BCS (Smith & Nephew, Memphis, TN, USA), was created to approximate normal knee kinematics [9] and was described as a guided motion TKA. The BCS knee system generates a tibial internal rotation during knee flexion by the guidance of the surface geometry and two cam-post mechanisms that substitute for the anterior and posterior cruciate ligaments. Inui et al. [10]. have reported that the amount of intraoperative tibial internal rotation between 30°, 60°, and 90° flexion and maximum flexion were correlated with improvement of postoperative PROMs following BCS TKA. Although studies have reported the relationship between the tibial rotational angle and intraoperative soft tissue balance in CR or PS TKA [7, 8], only one study has evaluated the effect of soft tissue balance on tibial internal rotation in BCS TKA [11]. Furthermore, although studies have shown that both medial and lateral soft tissue balances are important for good tibial internal rotation [7, 8], no study has evaluated the effects of soft tissue balance at medial or lateral compartments separately on tibial internal rotation in BCS TKA.

The authors hypothesized that both medial and lateral soft tissue balances influence rotational knee kinematics in BCS TKA. The purpose of this study was to retrospectively investigate the relationship between medial or lateral component gaps and rotational knee kinematics in BCS TKA.

## Materials and Methods

The institutional review board of the authors’ institution approved this study, and all patients who participated provided written informed consent.

From February 2016 to October 2019, 226 knees underwent primary TKA using Journey II BCS at the authors’ institution. In this study, the inclusion criteria were the following: (1) knees with varus deformity, (2) osteoarthritis of the knee, (3) the use of the image-free navigation system (Precision N; Stryker Orthopedics, Mahwah, NJ, USA), (4) intraoperative component gap was measured, and (5) intraoperative tibial rotational angle was measured. The knees which did not meet the inclusion criteria were excluded in this study. There were 10, 6, 10, 34, and 8 knees which were excluded in this study due to the first, second, third, fourth, and fifth inclusion criteria, respectively. One hundred fifty-eight knees met the inclusion criteria and thus were included in this study. Their characteristics and preoperative variables were shown in Table 1. All the procedures were performed using the same surgical technique by five knee surgeons. A senior surgeon (HI) participated in all procedures as either the chief surgeon or first assistant.

**Table 1:** Patient characteristics and preoperative variables

Age (years)	72.7 ± 8.6
Gender (female/male)	137/21
BMI (kg/m^2^)	26.7 ± 4.1
Preoperative range of motion	
Maximum extension (degrees)	− 9.5 ± 7.2
Maximum flexion (degrees)	118.3 ± 15.4
Preoperative HKA angle (degrees)	169.4 ± 5.7

Data are expressed as mean ± standard deviation

*BMI* Body mass index, *HKA* Hip–knee–ankle

### Surgical procedure

All patients underwent TKA using a paramedian approach, and the patella was not everted. The medial soft tissues were minimally released for bone resection. The balancing techniques focused on medial compartment stability [12, 13]. The distal femur and proximal tibia were osteotomized through the navigation system. Femoral alignment was aimed at a placement of 90° to the mechanical axis in the frontal plane and 4° of flexion in the sagittal plane. For the tibia, alignment was aimed at 90° to the mechanical axis in the frontal plane and 3° of posterior slope in the sagittal plane. The extension and flexion gaps were measured using a ligament tensioner, and the amount of posterior femur resection was adjusted to make the extension and flexion gaps of the medial compartment equal to acquire medial joint stability. Femoral rotation was determined as being parallel to the surgical epicondylar axis, allowing residual lateral ligamentous laxity [12, 13]. Tibial rotational alignment was determined using the range of motion (ROM) technique in which the knee was put through a full range of flexion and extension, allowing the tibial trial to orient itself to the best position relative to the femoral component and reducing component rotational mismatch [14]. The thickness of the polyethylene insert was determined to acquire intraoperative 0° flexion of the knee and medial compartment stability at 0° flexion.

### Intraoperative gap measurement

After these procedures, the extension and flexion gaps between the osteotomized surfaces were measured twice by the chief surgeon using the same ligament tensioner with a distraction force of 80 N for each compartment, and the averages were used. The mean (± standard deviation [SD]) joint gaps at extension and flexion were 22.1 ± 1.7 mm and 22.5 ± 1.9 mm, respectively, in the medial compartment and 24.2 ± 2.2 mm and 23.8 ± 2.4 mm, respectively, in the lateral compartment.

After evaluating the soft tissue balance between the osteotomized surfaces, the tensor device was put on the osteotomized surface of the tibia by placing the femoral trial component and reducing the patellofemoral joint. The tensor device consisted of three parts: upper compartment-specific plates, a lower platform plate, and an extra-articular main body [12]. The upper plates had identical shapes to that of the medial and lateral compartments of the polyethylene trial surface of the Journey II BCS system. This device was designed to allow surgeons to measure every millimeter of the joint component gaps of medial and lateral compartments. Using this tensor device, the component gaps of medial and lateral compartments were assessed at 0°, 30°, 60°, and 90° flexion measured using the navigation system with a joint distraction force of 80 N for each compartment. The medial or lateral joint laxities, which were defined as the value of component gap minus the selected thickness of the tibial component, were evaluated.

### Intraoperative tibial rotational angle evaluation

The tibial rotational angles after implantation at 0°, 30°, 60°, 90°, and maximum flexion were obtained for each patient using the navigation kinematic data during the motion cycles from 0° flexion to maximum flexion. These navigation kinematic data were evaluated three times, twice by the chief surgeon and once by the first assistant, and the averages were used. Among these rotational kinematic data, the following four parameters were evaluated while considering previous studies [7, 8, 10]: (1) the rotational angular difference between 0° flexion and maximum flexion (RAD 0), (2) the rotational angular difference between 30° flexion and maximum flexion (RAD 30), (3) the rotational angular difference between 60° flexion and maximum flexion (RAD 60), (4) the rotational angular difference between 90° flexion and maximum flexion (RAD 90). The tibial internal rotation relative to the femur was defined as a positive value.

### Postoperative evaluation

Postoperative ROM of the knee was measured at 1 year postoperatively. The postoperative lower limb alignment was obtained using full length standing radiographic images. Additionally, the component positions were postoperatively measured as follows: the femoral component positions relative to the femur with varus/valgus angles and flexion/extension angles, and the tibial component positions relative to the tibia with varus/valgus angles and anteriorly/posteriorly sloped angles [15]. The rotational alignment of the femoral and tibial components was evaluated using computed tomography images. The rotational femoral component angle was defined as the angle between the line of the anterior cutting surface and the surgical epicondylar axis. The rotational tibial component angle was defined as the angle between the line connecting the medial border of the tibial tuberosity to the center of the posterior concavity of the tibial component and the line passing through the anteroposterior center of the tibial component [16]. Varus, flexion, and external rotation for the femoral component and varus, posteriorly sloped, and external rotation for the tibial component were defined as positive values.

### Statistical analyses

According to a previous study which has shown that intraoperative joint laxities in BCS TKA were nearly constant at 2.8 ± 1.6 mm in the medial compartment and 3.3 ± 2.3 mm in the lateral compartment [17], the intraoperative joint laxities were firstly divided into two groups in the medial or lateral compartments at 0°, 30°, 60°, and 90° flexion, respectively. The groups according to the medial compartment were as follows: Group M-stable (medial joint laxity of ≤ 2 mm) and Group M-loose (medial joint laxity of ≥ 3 mm). The groups according to the lateral compartment were as follows: Group L-stable (lateral joint laxity of ≤ 3 mm) and Group L-loose (lateral joint laxity of ≥ 4 mm). Finally, the knees enrolled in this study were divided into four groups based on the combination of the medial and lateral joint laxities at 0°, 30°, 60°, and 90° flexion, respectively: Group A (M-stable and L-stable), Group B (M-stable and L-loose), Group C (M-loose and L-stable), and Group D (M-loose and L-loose). Among the 158 knees in this study, the numbers at 0°, 30°, 60°, 90° flexion between Group A, B, C, and D were shown in Tables 2.

**Table 2:** The numbers at each angle in Group A, B, C, and D

	Group A	Group B	Group C	Group D
0° flexion	130 knees	26 knees	1 knee	1 knee
30° flexion	41 knees	52 knees	10 knees	55 knees
60° flexion	49 knees	39 knees	19 knees	51 knees
90° flexion	44 knees	29 knees	18 knees	67 knees

Statistical analyses were performed using the statistical software EZR (version 1.31; Saitama Medical Center, Jichi Medical University, Saitama, Japan) [18]. An analysis of variance test was used to compare RAD 0, RAD 30, RAD 60, and RAD 90 between the four groups at each angle. All significance tests were two-tailed, and a significance level of *p* < 0.05 was used for all tests. For post hoc analyses, Tukey's honestly significant difference tests were conducted. Interclass and intraclass coefficient values of the intraoperative tibial rotational angle evaluated using the navigation system were > 0.80, indicating excellent reliability as well as previous report [10, 19]. The estimated sample size was 132 (1 − β = 0.80, α = 0.05) according to the statistical power analysis using G*Power (version 3.1.9.4, Heinrich Heine University, Düsseldorf, Germany) [20] Post hoc power analyses were adequate (> 0.80) except for the comparisons at 0° flexion; therefore, the authors investigated the comparisons between tibial rotational angles and joint laxities at 30°, 60°, and 90° flexion, respectively. The data were shown as the mean ± SD.

## Results

The mean medial and lateral joint laxities at each angle were shown in Table 3. The medial joint laxity was significantly smaller than the lateral joint laxity at each angle (*p* < 0.05 at each angle). The intraoperative maximum flexion angles which were measured using the navigation system were shown in Table 4. No statistical difference was found between the four Groups at each angle.

**Table 3:** Intraoperative medial or lateral joint laxity at each angle

	medial	lateral	*P* value
0° flexion	− 0.5 ± 1.4 mm	1.4 ± 2.2 mm	< 0.01
30° flexion	2.2 ± 1.6 mm	4.8 ± 2.7 mm	< 0.01
60° flexion	2.4 ± 1.7 mm	4.3 ± 2.7 mm	< 0.01
90° flexion	2.9 ± 1.6 mm	4.4 ± 2.6 mm	< 0.01

Data are expressed as mean ± standard deviation

**Table 4:** Intraoperative maximum flexion angle measured using the navigation system in Group A, B, C, and D

	Group A	Group B	Group C	Group D	*P* value
30° flexion (degrees)	135.5 ± 6.4	135.2 ± 5.7	136.0 ± 7.4	134.5 ± 6.1	0.85
60° flexion (degrees)	135.4 ± 7.1	135.5 ± 5.4	136.2 ± 6.3	134.1 ± 5.6	0.53
90° flexion (degrees)	135.3 ± 6.9	134.4 ± 5.6	135.8 ± 5.7	135.1 ± 6.0	0.89

**Fig. 1:**
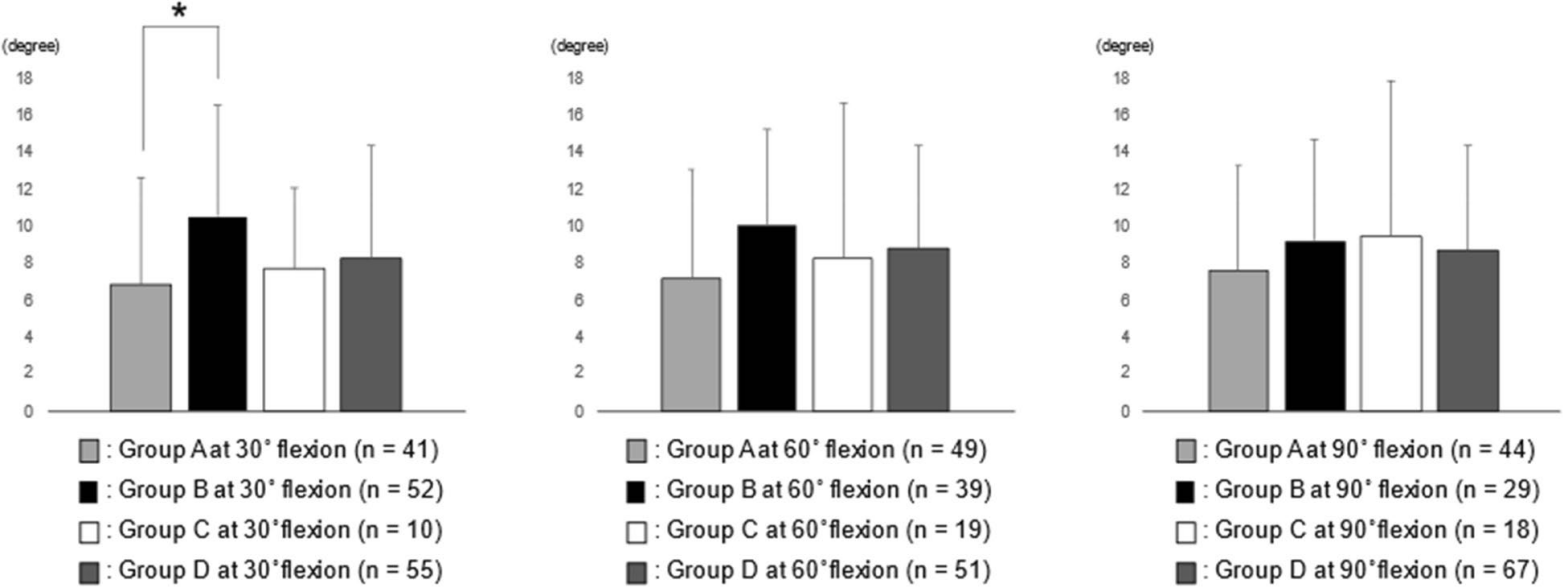
The analysis between RAD 0 and Group A, B, C, and D. RAD 0 in Group B at 30° flexion was significantly larger than that in Group A at 30° flexion (*p < 0.05). RAD 0, the rotational angular difference between 0° flexion and maximum flexion

The analysis between RAD 0 and Group A, B, C, and D were shown in Fig. 1. RAD 0 in Group B at 30° flexion was significantly larger than that in Group A at 30° flexion. No statistical correlation was observed between RAD 0 and the joint laxities at 60° and 90° flexion.

**Fig. 2:**
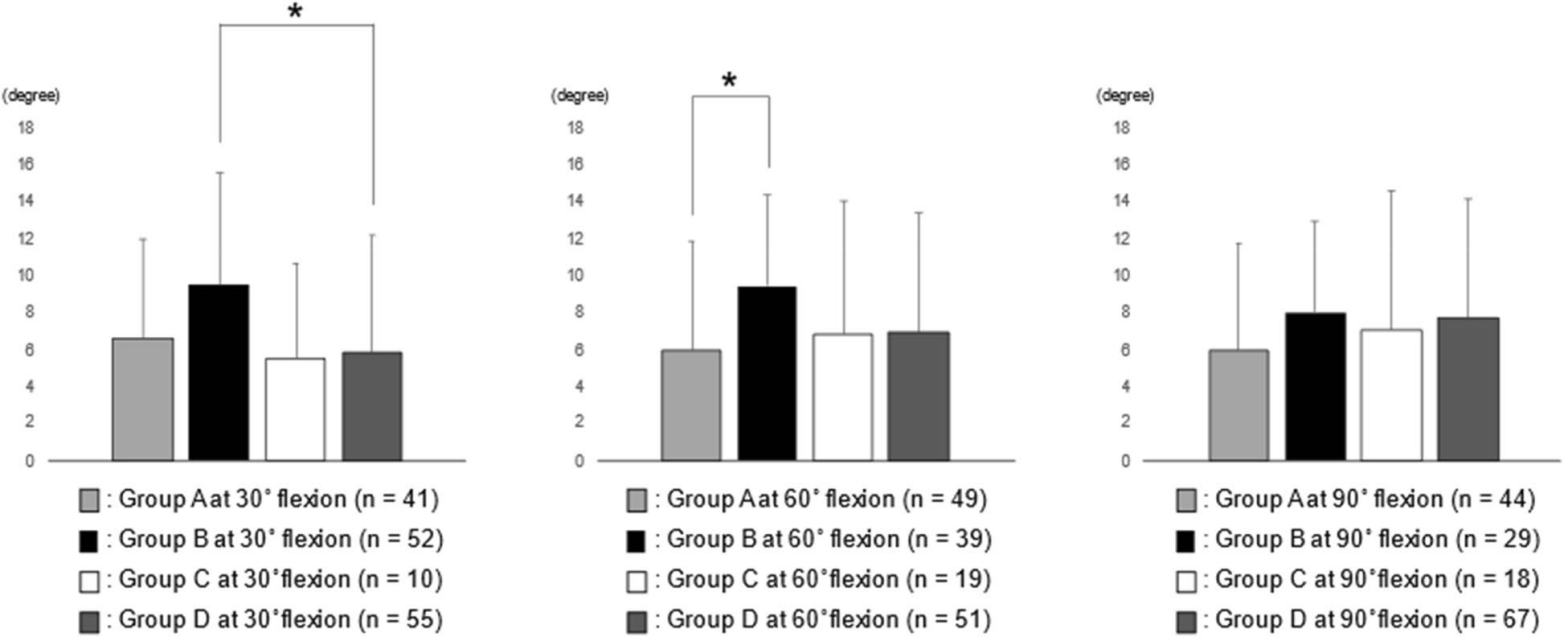
The analysis between RAD 30 and Group A, B, C, and D. RAD 30 in Group B at 30° flexion was significantly larger than that in Group D at 30° flexion (*p < 0.05). RAD 30 in Group B at 60° flexion was significantly larger than that in Group A at 60° flexion (*p < 0.05). RAD 30, the rotational angular difference between 30° flexion and maximum flexion

The analysis between RAD 30 and Group A, B, C, and D were shown in Fig. 2. RAD 30 in Group B at 30° flexion was significantly larger than that in Group D at 30° flexion. RAD 30 in Group B at 60° flexion was significantly larger than that in Group A at 60° flexion. No statistical correlation was observed between RAD 30 and the joint laxities at 90° flexion.

**Fig. 3:**
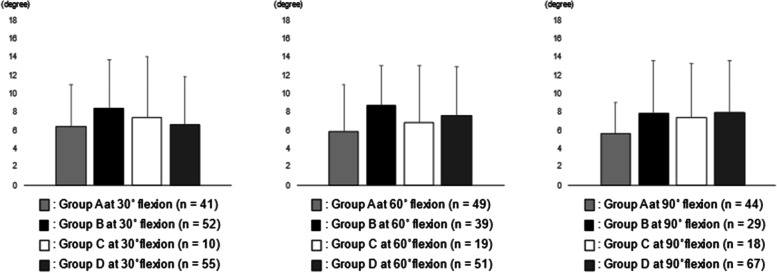
The analysis between RAD 60 and Group A, B, C, and D. No statistical correlation was observed between RAD 60 and the joint laxities at each angle. RAD 60, the rotational angular difference between 60° flexion and maximum flexion

The analysis between RAD 60 and Group A, B, C, and D were shown in Fig. 3. No statistical correlation was observed between RAD 60 and the joint laxities at each angle.

**Fig. 4:**
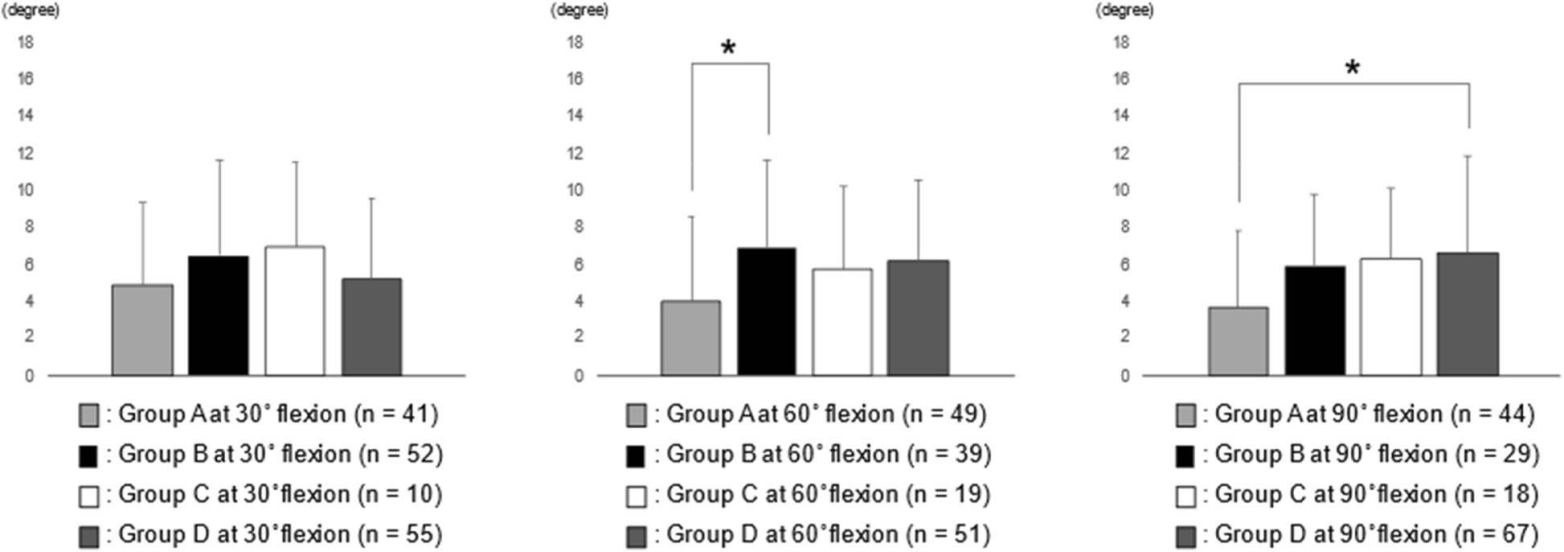
The analysis between RAD 90 and Group A, B, C, and D. RAD 90 in Group B at 60° flexion was significantly larger than that in Group A at 60° flexion (*p < 0.05). RAD 90 in Group D at 90° flexion was significantly larger than that in Group A at 90° flexion (*p < 0.05). RAD 90, the rotational angular difference between 90° flexion and maximum flexion

The analysis between RAD 90 and Group A, B, C, and D were shown in Fig. 4. The RAD 90 in Group B at 60° flexion was significantly larger than that in Group A at 60° flexion. The RAD 90 in Group D at 90° flexion was significantly larger than that in Group A at 90° flexion. No statistical correlation was observed between RAD 90 and the joint laxities at 30° flexion.

The postoperative evaluations including ROM of the knee, lower limb alignment, and femoral and tibial component positions were shown in Tables 5, 6 and 7. Femoral varus angle in Group B at 30° flexion was significantly smaller than that in Group D at 30° flexion. Femoral external rotational angle in Group B at 90° flexion was significantly smaller than those in Group C and Group D at 90° flexion. No statistical differences were found at postoperative ROM, lower limb alignment, and tibial component position.

**Table 5:** Postoperative range of motion of the knee and radiographic evaluations in Group A, B, C, and D at 30° flexion

	Group A	Group B	Group C	Group D	*P* value
Range of motion at 1 year postoperatively					
Maximum extension (degrees)	− 0.5 ± 2.5	− 0.4 ± 1.7	− 0.5 ± 1.6	− 1.1 ± 1.9	0.40
Maximum flexion (degrees)	125.2 ± 10.9	122.1 ± 16.2	127.0 ± 3.5	122.4 ± 10.1	0.48
Postoperative HKA angle (degrees)	179.2 ± 2.1	179.8 ± 2.1	179.3 ± 1.4	178.8 ± 2.0	0.08
Femoral varus angle (degrees)	0.7 ± 1.5	0.1 ± 1.5	0.1 ± 2.0	1.0 ± 2.0	0.04^a^
Femoral flexion angle (degrees)	4.5 ± 2.2	4.7 ± 2.6	5.1 ± 3.7	4.5 ± 3.2	0.91
Femoral external rotational angle (degrees)	0.2 ± 1.5	− 0.1 ± 1.8	0.9 ± 0.9	0.1 ± 1.8	0.38
Tibial varus angle (degrees)	0.2 ± 1.3	0.1 ± 1.2	0.4 ± 1.0	0.2 ± 1.3	0.88
Tibial posterior slope angle (degrees)	3.1 ± 1.7	3.2 ± 1.6	3.4 ± 11.7	3.0 ± 1.6	0.86
Tibial external rotational angle (degrees)	2.7 ± 5.0	4.2 ± 3.5	3.6 ± 3.6	2.6 ± 4.2	0.20

Data are expressed as mean ± standard deviation

*HKA* Hip–knee–ankle

^a^ Statistically significant (Group B vs Group D)

**Table 6:** Postoperative range of motion of the knee and radiographic evaluations in Group A, B, C, and D at 60° flexion

	Group A	Group B	Group C	Group D	*P* value
Range of motion at 1 year postoperatively					
Maximum extension (degrees)	− 0.8 ± 2.3	− 0.5 ± 2.2	− 0.7 ± 1.7	− 0.7 ± 1.8	0.90
Maximum flexion (degrees)	124.5 ± 10.9	122.1 ± 15.0	124.6 ± 7.6	122.8 ± 13.2	0.79
Postoperative HKA angle (degrees)	179.3 ± 1.8	179.8 ± 2.3	178.9 ± 2.0	179.0 ± 2.0	0.30
Femoral varus angle (degrees)	0.5 ± 1.5	0.3 ± 1.6	0.6 ± 2.1	0.8 ± 2.0	0.54
Femoral flexion angle (degrees)	4.8 ± 2.4	4.4 ± 2.3	4.4 ± 3.0	4.6 ± 3.4	0.95
Femoral external rotational angle (degrees)	0.4 ± 1.8	− 0.4 ± 1.8	0.4 ± 1.0	0.1 ± 1.8	0.14
Tibial varus angle (degrees)	0.3 ± 1.3	− 0.1 ± 1.3	0.3 ± 1.2	0.1 ± 1.2	0.60
Tibial posterior slope angle (degrees)	3.1 ± 1.4	3.3 ± 1.8	2.7 ± 2.0	3.2 ± 1.6	0.56
Tibial external rotational angle (degrees)	2.9 ± 4.5	3.4 ± 3.0	3.1 ± 4.6	3.2 ± 4.6	0.95

Data are expressed as mean ± standard deviation

*HKA* hip–knee–ankle

**Table 7:** Postoperative range of motion of the knee and radiographic evaluations in Group A, B, C, and D at 90° flexion

	Group A	Group B	Group C	Group D	*P* value
Range of motion at 1 year postoperatively					
Maximum extension (degrees)	− 1.2 ± 2.4	− 0.8 ± 2.2	− 0.2 ± 1.9	− 0.4 ± 1.6	0.17
Maximum flexion (degrees)	122.1 ± 11.4	122.2 ± 15.1	123.1 ± 17.3	124.7 ± 10.4	0.68
Postoperative HKA angle (degrees)	179.0 ± 1.9	179.8 ± 2.4	179.1 ± 2.0	179.3 ± 2.0	0.53
Femoral varus angle (degrees)	0.7 ± 1.6	0.0 ± 1.8	0.4 ± 1.7	0.7 ± 1.8	0.28
Femoral flexion angle (degrees)	4.7 ± 2.2	4.2 ± 2.3	5.2 ± 3.3	4.5 ± 3.1	0.65
Femoral external rotational angle (degrees)	0.2 ± 1.4	− 0.8 ± 1.7	0.8 ± 1.3	0.2 ± 1.9	0.01^b^
Tibial varus angle (degrees)	0.2 ± 1.3	0.2 ± 1.4	0.3 ± 0.9	0.0 ± 1.2	0.66
Tibial posterior slope angle (degrees)	3.3 ± 1.5	3.4 ± 1.9	2.3 ± 1.4	3.1 ± 1.6	0.12
Tibial external rotational angle (degrees)	3.0 ± 4.4	3.0 ± 3.7	4.1 ± 4.7	3.2 ± 4.2	0.79

Data are expressed as mean ± standard deviation

*HKA* hip–knee–ankle

^b^ statistically significant (Group B vs Group C, Group B vs Group D)

## Discussion

The most important finding in this study was that both medial and lateral soft tissue balances influenced the intraoperative rotational knee kinematics in BCS TKA. The combinations of the medial joint laxity of ≤ 2 mm and the lateral joint laxity of ≥ 4 mm at 30° and 60° flexion and the medial joint laxity of ≥ 3 mm and lateral joint laxity of ≥ 4 mm at 90° flexion were important in achieving a better tibial internal rotation.

Medial joint stability was previously reported to be important for achieving good rotational knee kinematics following conventional TKA. Wada et al. [21] have investigated the influence of medial collateral ligament (MCL) release on rotational knee kinematics in frozen cadaveric knee undergoing PS TKA. They argued that extensive MCL release reduced the amount of tibial internal rotation during knee flexion. Similarly, substantial medial release including semimembranosus tendon also reported to reduce tibial internal rotation during flexion in CR TKA [22]. Nakamura et al. [23] have evaluated the relationship between the postoperative flexion gap measured using axial radiography and in vivo knee kinematics using fluoroscopy in cruciate-substituting TKA and concluded that medial joint laxity caused abnormal knee kinematics. As for the relationship between intraoperative component gap and intraoperative tibial rotational angle, the amount of tibial internal rotation was negatively correlated with the medial component gap at 60° flexion in PS TKA [7]. In this study, the intraoperative medial compartment at 0° flexion was stabilized by the surgical procedure focused on medial compartment stability [12, 13] and the manner of selection of polyethylene insert thickness (Table 3). Furthermore, RAD 30 in Group B at 30° flexion was significantly larger than that in Group D at 30° flexion in BCS TKA (Fig. 2). In other words, in the case of loosening of the lateral compartment, medial joint stability at midflexion was positively correlated with intraoperative tibial internal rotation. Increasing joint laxity at midflexion was reported to negatively influence postoperative PROMs [24], and from the results of this study, medial joint stability at midflexion was also important for achieving good tibial internal rotation in BCS TKA.

Regarding lateral soft tissue balance, lateral joint laxities in a normal knee have reported to be greater than medial joint laxities in both extension and flexion [25, 26]. Furthermore, lateral joint laxity has reported to positively correlate with postoperative flexion angle [27] or PROMs [28] following conventional TKA. As for the relationship between intraoperative component gap and intraoperative tibial rotational angle, the amount of tibial internal rotation was positively correlated with the lateral component gap at 60°, 90°, and 120° flexion in CR TKA [22]. This study showed that RAD 0 in Group B at 30° flexion was significantly larger than that in Group A at 30° flexion (Fig. 1), and RAD 30 and RAD 90 in Group B at 60° flexion were significantly larger than those in Group A at 60° flexion (Figs. 2 and 3). In other words, in the case of stabilizing of the medial compartment, lateral joint laxity at midflexion was positively correlated with intraoperative tibial internal rotation. As well as conventional TKA, lateral joint laxity was thought to be important for achieving good tibial internal rotation in BCS TKA. However, some reports have shown that lateral joint laxity has somewhat negative effects on PROMs [29]; therefore, further investigation to clarify the most appropriate amount of lateral joint laxity in achieving better clinical results in BCS TKA is required.

In this study, while RAD 30 in Group B at 30° flexion was significantly larger than that in Group D at 30° flexion (Fig. 2), RAD 90 in Group D at 90° flexion was significantly larger than that in Group A at 90° flexion (Fig. 4). In other words, medial and lateral joint laxities at 90° flexion was positively correlated with intraoperative tibial internal rotation. Inui et al. [11] reported that relatively loose flexion osteotomy gap showed good intraoperative tibial internal rotation in BCS TKA. As well as previous study [11], too tight flexion gap may need to be prevented for good intraoperative kinematics in BCS TKA.

Postoperative radiographic evaluations in this study revealed that the femoral varus angle in Group B at 30° flexion was significantly smaller than that in Group D at 30° flexion, and femoral external rotational angle in Group B at 90° flexion was significantly smaller than those in Group C and Group D at 90° flexion. In general, increasing femoral valgus angle cause decreasing medial component gap and increasing lateral component gap. Furthermore, increasing femoral internal rotational angle theoretically cause decreasing medial component gap and increasing lateral component gap. Femoral implantation with increasing femoral valgus angle and internal rotational angle may result in medial joint stability and lateral joint laxity and may possibly achieve good tibial internal rotation in BCS TKA. However, the femoral alignment in the frontal plane in the Journey II BCS system is 3° valgus with respect to the orthogonal to the mechanical axis for restoring the natural frontal inclination of the joint line; therefore, additional valgus alignment of the femoral component might cause an excessive stress of the polyethylene insert. In addition, internal rotation of the femoral component alignment may cause some problems on patellofemoral joint tracking. Therefore, the appropriate alignment of femoral and tibial components cannot be identified only from this study. Further investigation about the relationship between the component alignments, knee kinematics, and clinical results is needed.

This study has several limitations. First, this was a retrospective study. Second, the sample size was relatively small, although post hoc power analyses were adequate except for the comparisons of medial or lateral joint laxities at 0° flexion; therefore, the authors cannot argue the clinically meaningful significance for the comparison of medial or lateral joint laxities at 0° flexion from this study. Further investigation with large cohort is needed in the future. Third, the authors only investigated varus deformities; therefore, the results of this study cannot be generalized and applied to valgus deformities. Fourth, although the upper plates of the tensor device had identical shapes of the polyethylene surface in the medial and lateral compartments of the Journey II BCS system, they did not include the tibial post construct. The tibial post deficit may influence anterior–posterior position of the femoral and tibial components and change the intraoperative soft tissue balance or tibial rotational angle. Fifth, as well as previous studies [7, 8, 10, 11, 22, 23], the authors only evaluated intraoperative tibial rotational angle using the navigation system; therefore, the results of this study cannot prove to be reflected the intraoperative pivot pattern. Furthermore, anterior–posterior, medial–lateral, and superior-inferior translation were not evaluated. The relationship between three-dimensional intraoperative knee kinematics including pivot pattern and intraoperative medial and lateral joint laxities should be examined. Sixth, some significant relationships between the intraoperative joint laxities and intraoperative tibial rotational angle were revealed from this study, however, postoperative ROM of the knee were not statistically different between the four groups. The influence of the differences of intraoperative tibial rotational angle on postoperative clinical results were unclear from this study. Recently, the discordance between the clinical outcomes assessed by surgeons and patients has been reported [30]; therefore, PROMs is important for evaluating postoperative clinical outcomes of TKA, as well as the postoperative ROM and radiographic evaluations. The authors believe that the results from this study will help an improvement of clinical results of BCS TKA, however, postoperative PROMs should also be examined in the future. Furthermore, although the femoral alignments in the frontal and axial plane may possibly associate with intraoperative tibial rotation, the authors cannot argue the ideal alignment of femoral and tibial components or appropriate surgical procedure from this study because of the concern about the negative effects of adjustment of the femoral component alignment.

## Conclusions

The relationships between intraoperative joint laxity and intraoperative tibial rotational angle in BCS TKA were investigated in this study. The combinations of the medial joint laxity of ≤ 2 mm and the lateral joint laxity of ≥ 4 mm at 30° and 60° flexion and the medial joint laxity of ≥ 3 mm and lateral joint laxity of ≥ 4 mm at 90° flexion were important in achieving a better tibial internal rotation. Surgeons should pay attention to the importance of medial joint stability at midflexion and lateral joint laxities at midflexion and 90° flexion to achieve good tibial internal rotation in BCS TKA.

## Data Availability

The datasets generated and analyzed during the current study are not publicly available due to privacy concern of participants but are available from the corresponding author on reasonable request.

## Abbreviations

TKA: Total knee arthroplasty
PROM: Patient-reported outcome measure
CR: Cruciate-retaining
PS: Posterior-stabilized
BCS: Bi-cruciate stabilized
RAD: Rotational angular difference
MCL: Medial collateral ligament
